# Achieving the 95 95 95 targets for all: A pathway to ending AIDS

**DOI:** 10.1371/journal.pone.0272405

**Published:** 2022-08-04

**Authors:** Luisa Frescura, Peter Godfrey-Faussett, Ali Feizzadeh, Wafaa El-Sadr, Omar Syarif, Peter D. Ghys, on behalf of the 2025 testing treatment target Working Group

**Affiliations:** 1 Strategic Information Department, UNAIDS, Geneva, Switzerland; 2 London School of Hygiene and Tropical Medicine, London, United Kingdom; 3 ICAP at Columbia University, Mailman School of Public Health, New York, New York, United States of America; 4 Global Network of People Living with HIV (GNP+), Amsterdam, Netherlands; University of Pittsburgh, UNITED STATES

## Abstract

In December 2020, UNAIDS released a new set of ambitious targets calling for 95% of all people living with HIV to know their HIV status, 95% of all people with diagnosed HIV infection to receive sustained antiretroviral therapy, and 95% of all people receiving antiretroviral therapy to have viral suppression by 2025. Adopted by United Nations Member states in June 2021 as part of the new Political Declaration on HIV and AIDS, these targets, combined with ambitious primary prevention targets and focused attention to supporting enablers, aim to bridge inequalities in treatment coverage and outcomes and accelerate HIV incidence reductions by focusing on progress in all sub-populations, age groups and geographic settings. Here we summarise the evidence and decisions underpinning the new global targets.

## Introduction

At the 20^th^ International AIDS Conference in Melbourne, Australia, in 2014, UNAIDS launched new global targets for HIV treatment scale up. Known as the three 90’s (by 2020, 90% of all people living with HIV will know their HIV status; by 2020, 90% of all people with diagnosed HIV infection will receive sustained antiretroviral therapy (ART); by 2020, 90% of all people receiving ART will have viral suppression) they reflected a fundamental shift in the approach to HIV treatment, moving from a focus on the number of people accessing antiretroviral therapy towards case detection, and a cascade of services that seek to ensure that people living with HIV achieve viral suppression and its consequent individual and societal benefits [1,2].

The 90-90-90 targets have been widely adopted and largely successful in putting additional focus on the scale up of ART, similar to previous initiatives such as “3 by 5” which aimed to treat 3 million people in low- and middle-income countries (LMICs) [3] by 2005 and “15 by 15” which aimed to reach 15 million people with ART by 2015. Although the treatment goal established by “3 by 5” was reached after the deadline, it is credited with mobilizing the world to focus on expansion of access to treatment and establishing ART as an essential public health intervention, and subsequently the “15 by 15” target was met 9 months ahead of schedule. Although the ambition of the three 90’s has led to 27.5 million [26.5 million–27.7 million] people on ART at the end of 2020, [4] progress has been uneven across countries and population groups, and the impact on reducing new HIV infections has been more limited than projected by early modelling studies [5]. For example, a recent modelling study examining trends in knowledge of HIV-positive status from 2000–2019 in 40 countries in sub-Saharan Africa, found that although knowledge of HIV-positive status has increased substantially and steadily over the past decade, it remains lower among men, and among young people aged 15–24 years than among other groups [6].

The need to overcome these challenges to reach the 2030 target of ending AIDS as a public health threat as defined in the Agenda on Sustainable Development [7] has led to the elaboration of a new set of targets on testing and treatment for 2025.

This paper reviews the evidence and analysis which led to the 2025 global HIV testing and treatment targets and the new emphasis on addressing inequalities in access and outcomes. It lays out the analyses and arguments for achieving the 95-95-95 targets in each relevant sub-group of the population.

## Methods

Inputs to inform the new set of targets on testing and treatment were provided through technical consultations with the 2025 Testing and Treatment Target Working Group [7] (see acknowledgments). The data review included the collation of existing data and analyses on HIV incidence and prevalence from various sources across LMICs. The Working Group described progress against the 2020 targets of 90-90-90 and a) considered whether raising the targets was credible, b) reviewed the evidence of inequalities in treatment coverage and c) reviewed the evidence on the impact of treatment on HIV transmission. Data sources included modelled UNAIDS epidemiological estimates, population HIV-based assessments, and results of community randomized test and treat trials.

### Progress against the 90-90-90 targets and credibility of increasing target

UNAIDS publishes national estimates for each of the three 90’s using previously described models that incorporate the most recent data from each country [8,9]. These estimates are validated and approved by national HIV programmes. The most recent estimates were compared to data collected in large population-based HIV assessments in 11 countries with available data [10]. In addition, data from four large community randomized test and treat trials [11–15] were reviewed to understand changes in the 90-90-90 indicators. Lastly, new testing and treatment technologies and approaches to be anticipated in the next 5 years were considered, and are discussed in a separate paper in this collection [16].

### Evidence of disparities in testing, treatment and viral suppression

Existing evidence of testing and treatment indicators for key populations (gay men and other men who have sex with men, transgender people, sex workers, people who use drugs and incarcerated people) were reviewed.

The national modelled estimates, the population-based survey data and the data from the randomized trials were reviewed by age-group, sex and geography.

### Impact of treatment on epidemic transition

The ratio of the number of new HIV infections per year to the number of people living with HIV at the mid-point of that year (the Incidence:Prevalence ratio or IPR) provides a useful metric to quantify progress towards epidemic transition [17–19]. It provides a global benchmark by considering the replacement level for HIV infection: for an epidemic to decline, on average, there should be fewer than one new infection per person living with HIV over the course of their life. It assumes that a newly infected person may live on average for 33 years with HIV while on antiretroviral therapy. One new infection in this 33 year period, or the reciprocal of the average time a person lives with HIV following initial infection (1/33 or 0.03) would therefore constitute the threshold at which the HIV epidemic would be stable. If an IPR value less than 0.03 is maintained over time, an epidemic would eventually be eliminated. This measure of epidemic transition was discussed and proposed at a meeting convened by UNAIDS October 2017 [17] and has been included in UNAIDS reports [20,21] as well as several research papers [18,22] Data on incidence, prevalence and viral load suppression were extracted from UNAIDS estimates [21] and from published population-based surveys. The IPR and proportion of the population estimated to have unsuppressed HIV viral load were calculated for countries with available data in eastern and Southern Africa.

In epidemic settings where the majority of HIV transmissions was through heterosexual contact, the IPR can be adapted to incorporate an analysis by sex. The ratio of male incidence to female prevalence provides an estimate of the number of men who acquired HIV infection from a woman, while the ratio of female incidence to male prevalence provides an estimate of the number of women who acquired HIV infection from a man. This opposite-sex version of the IPR highlights the incidence in men which arises from the prevalence in women and vice versa.

Using cross-sex IPRs is therefore appropriate for settings where most new infections arise from heterosexual contact. Such analyses are not relevant in settings where same sex transmission is more common, either through men having sex with men, people of the same sex using drugs leading to parenteral transmission or, extremely rarely, through women having sex with women. For countries where most of the transmission is through heterosexual contact, the cross-sex IPR were compared for countries with an HIV prevalence greater than 2%.

Data from four large recent community randomized trials of interventions that aimed to increase coverage of HIV testing, linkage and treatment at population level were reviewed to explore the relationships between viral load suppression and incidence: Botswana Combination Prevention Project (BCPP) [15], HPTN 071—Population Effects of Antiretroviral Therapy to Reduce HIV Transmission (PopART) [13], Sustainable East Africa Research in Community Health (SEARCH) [14] and the HIV treatment as Prevention trial (ANRS) [12].

## Results

### Progress against the 90-90-90 targets and plausibility of increasing the targets

Global progress has been steady and at the end of 2020, 84% (67%-98%) of people living with HIV knew their HIV-positive status; among those who knew their status, 87% (67%-98%) were on treatment, and 90% (70%-98%) of those on treatment had achieved viral suppression [4]. In 2020, both Eswatini and Switzerland were estimated to have achieved over 86% viral suppression for all PLHIV (the target for viral suppression resulting as the product of 95-95-95) while six additional countries (Rwanda, Qatar, Botswana, Slovenia, Uganda, Malawi) had achieved the 90-90-90 targets by 2020 (see Table 1 in S1 Annex) [23].

Given that several countries have already reached the 90-90-90 target, with more expected to follow in the coming years, and the emergence of countries reaching coverage levels as high as 95% for testing, treatment and virologic suppression, the experts convened considered it credible to increase the 2020 targets. The range of geographical diversity, income status and epidemiology in the countries that have reached the 90-90-90 targets further highlighted the feasibility of setting ambitious targets for all countries, rather than setting different target levels for sets of countries with different characteristics.

**Fig 1:**
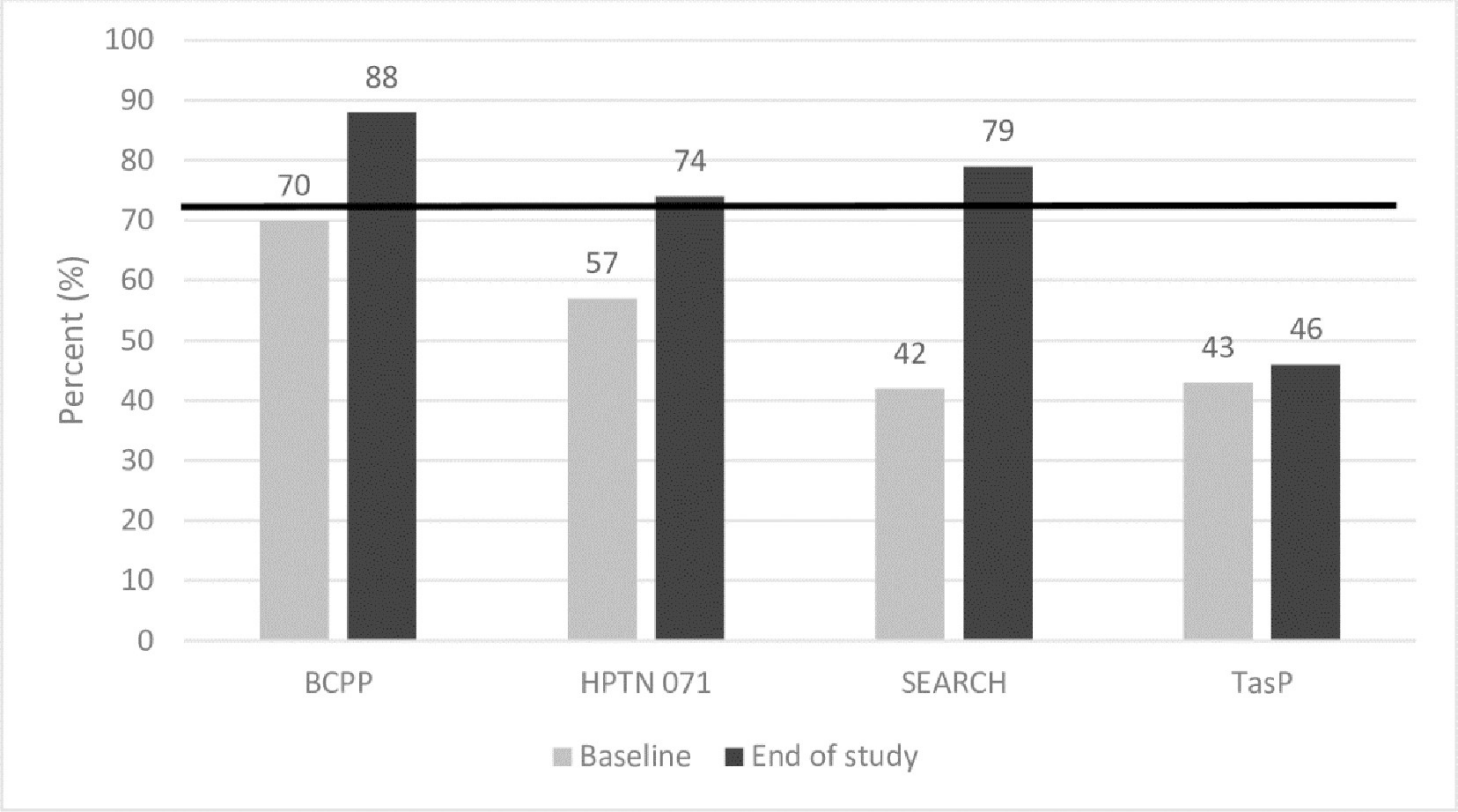
Universal test and treat (UTT) intervention arms: Baseline and end of study population‐level viral suppression. Source: Havlir, D., et al., *What do the Universal Test and Treat trials tell us about the path to HIVepidemic control?* J Int AIDS Soc, 2020. **23**(2): p. e25455.

Improvements in coverage observed across the combination community trials [11] (Fig 1) offered insights into implementation approaches that can inform efforts to achievement of the new targets, although considerable efforts in programme design, and implementation are needed to sustain progress. For example, community HIV-care Providers (CHiPs) played a key role in the provision of door-to-door HIV prevention services in the HPTN 071 trial [13]. Notably, HPTN 071, SEARCH, and BCPP trials met the 90-90-90 targets of achieving 73% population-level viral suppression among all PLHIV over the study period, although with inconsistent evidence of HIV incidence decline across the studies [11].

### Evidence of inequalities

**Fig 2:**
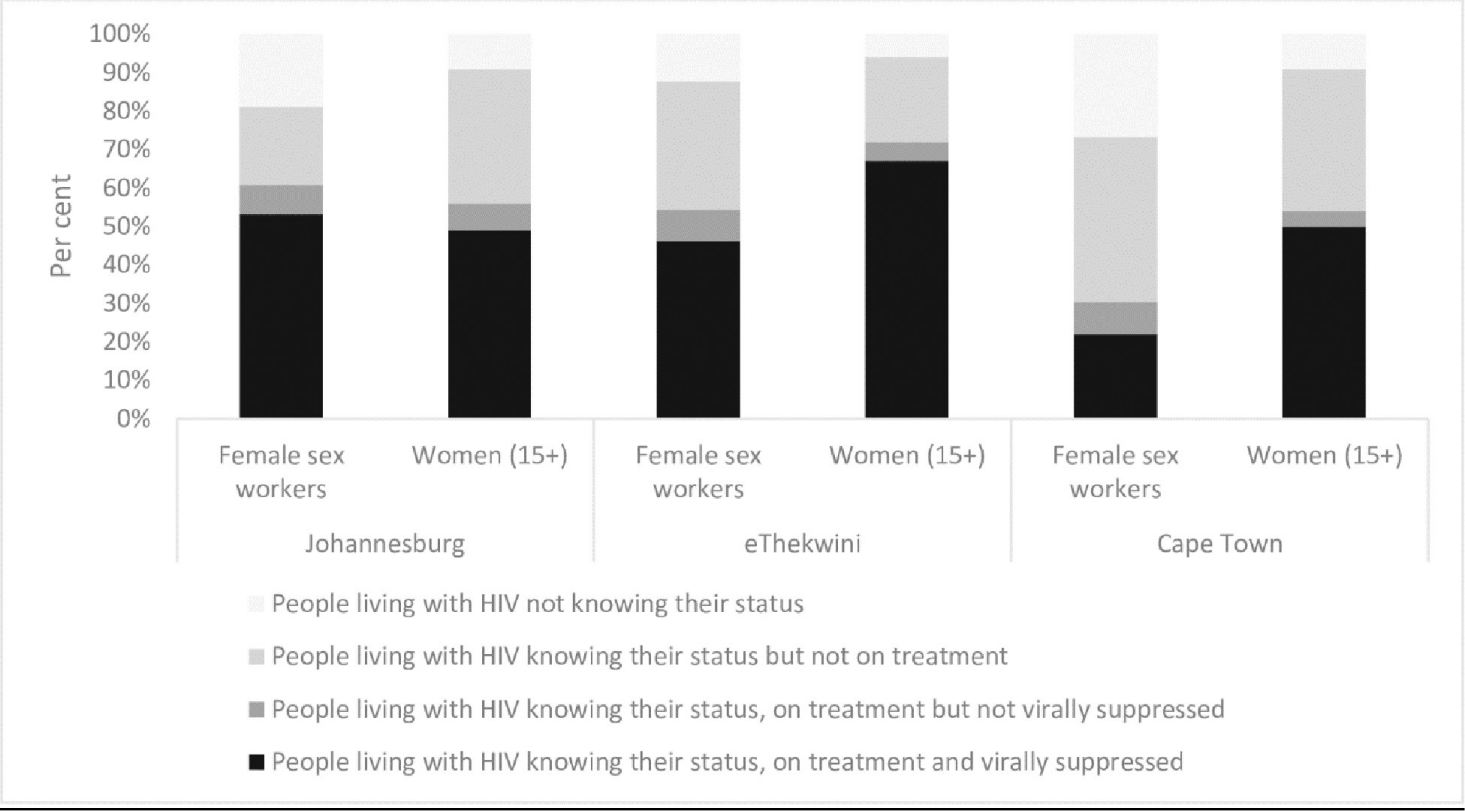
Knowledge of HIV status, treatment and viral suppression among female sex workers compared to the general population in South Africa, select districts, 2018. Source: South African Health Monitoring Survey, 2018; South Africa District HIV Estimates, 2017 (https://www.hivdata.org.za/).

**Fig 3:**
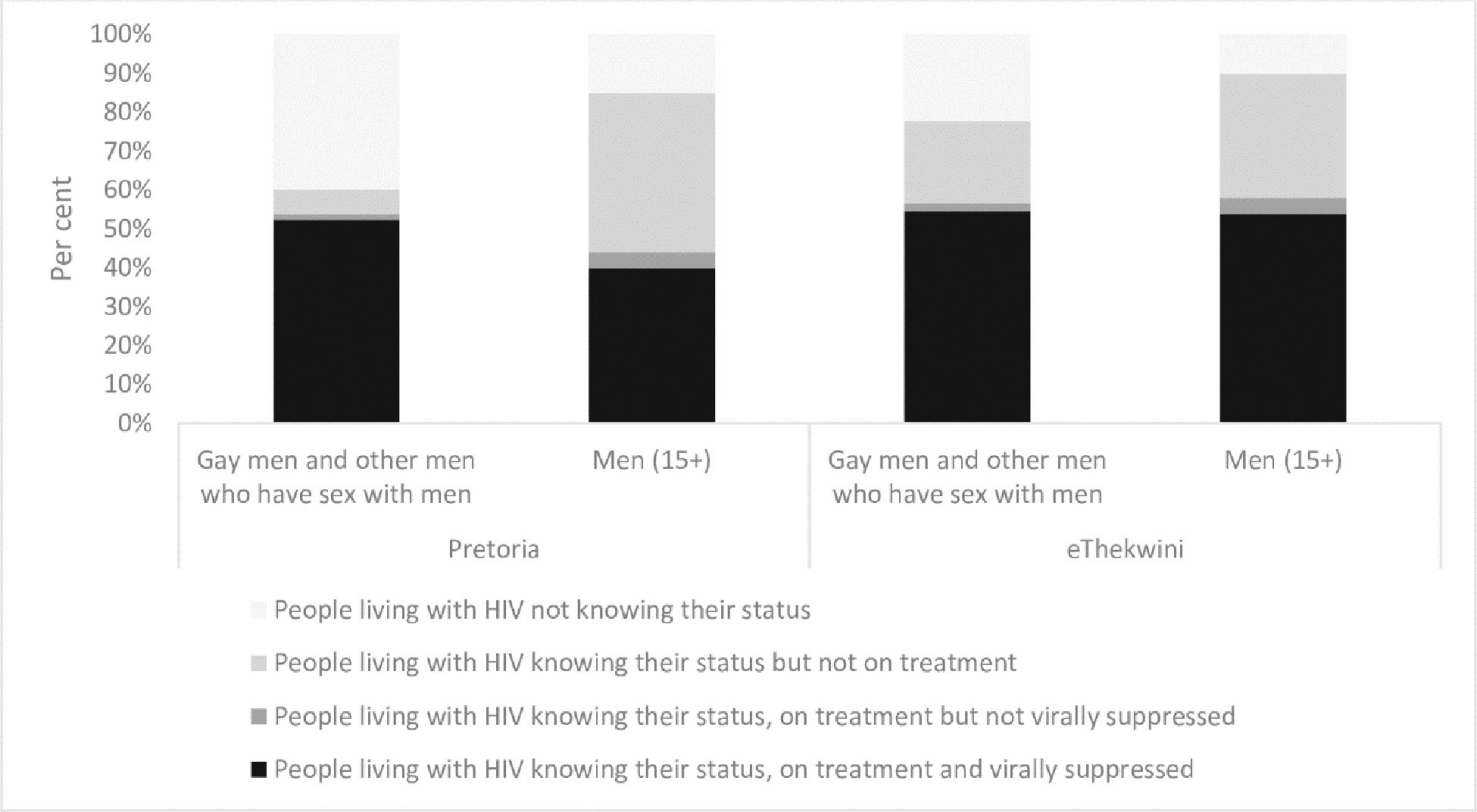
Knowledge of HIV status, treatment and viral suppression among gay men and other men who have sex with men compared to the general population in South Africa, select districts, 2018. Source: South African Health Monitoring Survey, 2018; South Africa District HIV Estimates, 2017 (https://www.hivdata.org.za/).

Although evidence on key populations is often limited, and existing data may be biased, evidence from district level data in South Africa shows that female sex workers, and men who have sex with men living with HIV were consistently less likely to know their HIV-positive status than the general population as noted in Figs 2 and 3 [4]. However, among those who were aware of their status, disparities in viral suppression were less marked.

**Fig 4:**
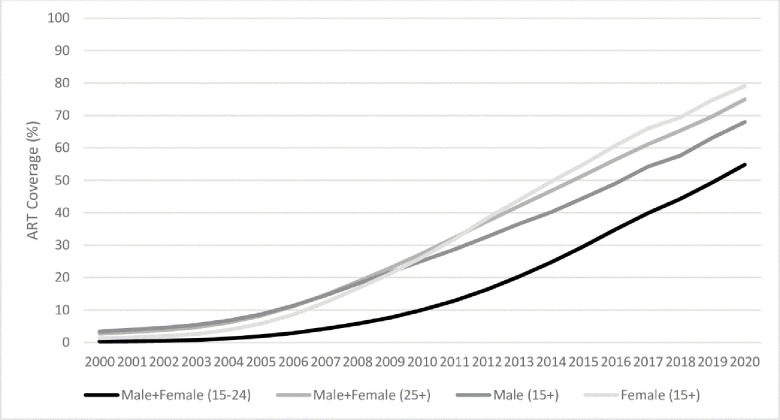
Percentage of adults accessing antiretroviral therapy by sex and age, global, 2000–2020. Source: UNAIDS Estimates, 2021.

Globally, important gaps in the achievement of the 90-90-90 targets also were shown for children, for men and young adults (aged 15–24 years), where treatment coverage among children living with HIV (54% (37%-69%) in 2020)) is well below the coverage for adults aged 15 and over (74%(57%-90%) and coverage among men ((68% (54%-83%)) is also lagging behind coverage for women ((79% (61%-95%)) [4]. In addition, ART coverage among young adults aged 15–24 in 2020 is estimated to be 55% compared to 75% among those over 25 years of age as noted in Fig 4 [21].

Similar gaps were observed in the community randomized trials. Although the first two of the three 90’s were reached overall in adults aged 15 years and older in the 7 intervention communities in Zambia and South Africa included in HPTN 071 (PopART trial), lower ART coverage for men and young people were observed. The proportion of women in Zambia and South Africa who knew their HIV status was 95% and 94% respectively, compared to men who reached 86% and 87% [13].

### Impact of treatment on epidemic transition

The all-gender incidence to prevalence ratio among adults has been declining in all regions since 2000 nearing an IPR threshold of 3% in Eastern and Southern Africa, West and central Africa, and western and central Europe and North America (see S2 Annex).

**Fig 5:**
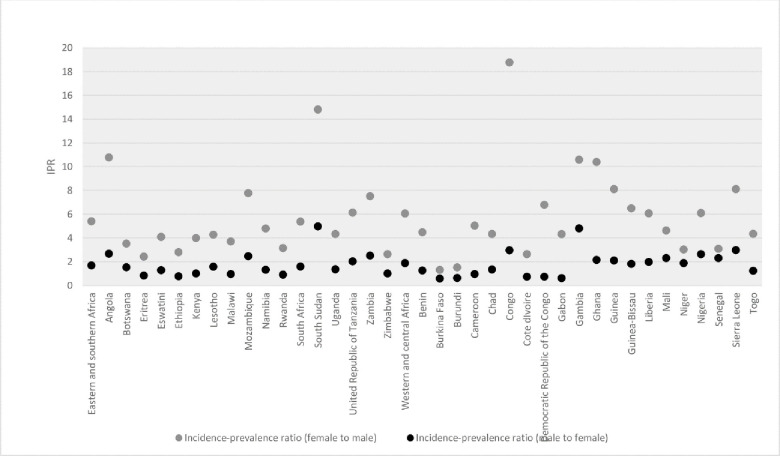
Cross-sex incidence-prevalence ratios in countries in sub-Saharan Africa, 2020. Source: UNAIDS Estimates, 2021.

Although there is considerable heterogeneity in the IPR among countries with an HIV prevalence greater than 2%, 2020 epidemiological estimates indicated that the female to male IPR in 2020 was consistently higher than the male to female IPR in all sub-Saharan African countries as noted in Fig 5.

**Fig 6:**
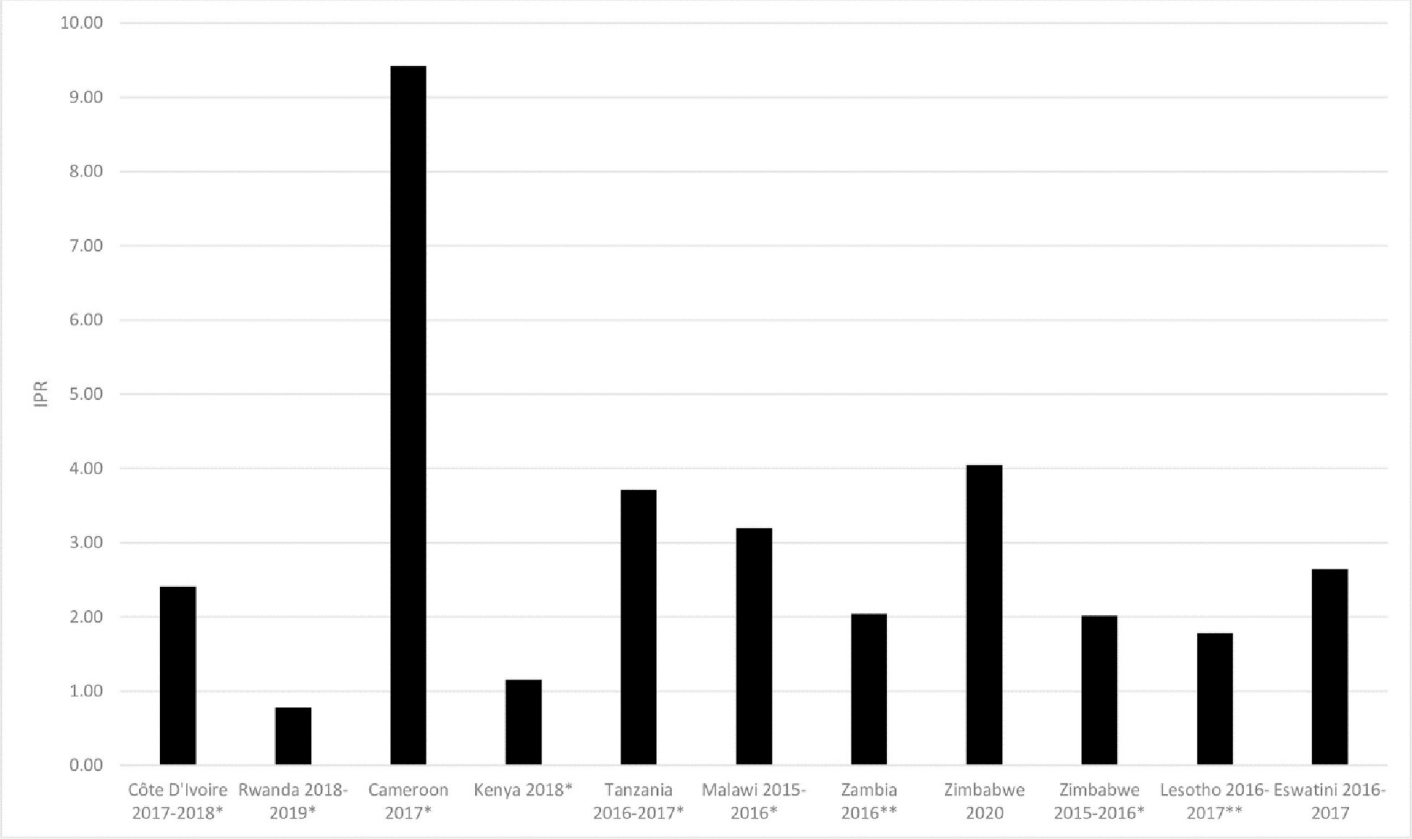
Cross-sex ratio of IPRs (male to female: female to male) in countries with available population-based survey data, sub-Saharan Africa. *15–64 **15–59. Source: ICAP PHIA.

This was also reflected in data from population-based surveys where in 10 of 11 countries in Sub-Saharan Africa where a survey was conducted and data were publicly available, the ratio of female HIV Incidence to male HIV prevalence was greater than the ratio of male HIV incidence to female HIV prevalence with a median of 2.41 (IQR: 1.9–3.45) as noted in Fig 6.

In addition, recent analysis that combined the data from community studies cited above showed a strong association between prevalence of viremia at community level and HIV incidence across all four trials. Specifically, HIV incidence increased by 0.07/100 person years for each 1% absolute increase in viremia [24]. Given the evidence that communities with higher viremia had higher HIV incidence, this finding supports the hypothesis that increasing coverage targets for testing and treatment would lead to reduction in HIV transmission at the population-level.

## Discussion

Overall, the global scale up of antiretroviral therapy since 2005 has seen the achievement of the 90-90-90 targets in 8 countries with diverse epidemics and geographies. This remarkable success has led to a renewed, ambitious vision to reach 95-95-95 testing and treatment targets by 2025 across all population groups and maintaining the current momentum in testing and ART scale up. The emphasis on making progress in testing and treatment coverage among all population groups seeks to address inequalities in achievement of treatment outcomes by age, sex, geography, and key populations and to optimize the effect of treatment on HIV incidence and individual health and well-being. However, reinvigorated investments in the HIV response in low- and middle-income countries (LMICs) will be needed to reach the ambitious new targets by 2025. Total expenditures for HIV testing and treatment will need to reach US$ 9.5 billion per year by 2025, up from US$ 8.3 billion which were spent in 2019 [25], even after accounting for efficiency gains such a reducing the costs for antiretrovirals.

However, inequalities in treatment coverage leave some population groups behind and vulnerable to ongoing HIV transmission. Evidence of sex disparities explored in this paper using IPRs from several sources reinforces the need to scale up treatment among men, and young people and to ensure that targets are met in all population groups. Transmission dynamics also re-emphasize the critical role of male to female transmission in perpetuating ongoing infections in the heterosexual epidemics of sub-Saharan Africa. Given that transmission from men to women is at least twofold higher than vice-versa, while the data indicate that there are many fewer men living with HIV than women in these high burden settings [21] the focus needs to be on providing tailored services that engage, test, support, link and treat men, young people, and key populations. Even in countries having achieved the 90 90 90 targets, the global or national average masks the underlying inequality which persists by geography and socioeconomic status. For example, a recent analysis of a population-based survey data in South Africa found that achievement of the 90 90 90 targets varied by geographic area with the first 90 target met in Free State Province (91.1%) only, while other provinces such as Northern Cape were lagging behind, particularly in farming communities (56.2%) [26]. Closing these gaps will require that countries employ a range of people-centered services tailored to the needs of the populations being served, including societal barriers that perpetuate stigma.

In addition, the effect of viral suppression on reducing incidence will only be fully realized when testing and treatment targets are achieved in all populations including for the above subgroups as well as in differing geographies [27,28]. This requires a focus on the individual, societal and structural barriers that certain populations face in accessing services and working with individuals and organizations representing such groups to overcome these barriers and to put in place concrete enablers [29].

Many of the data used in these analyses come from populations surveys and mathematical models, both with their associated limitations. However, these results are consistent with recent phylogenetic studies in the community trials which suggest that male to female transmission is around fourfold more common than female to male transmission [30–32] and highlight the need for targeted prevention, testing and treatment services to interrupt patterns of onwards HIV transmission. Achievement of viral load suppression has also been clearly linked to individual benefits for people living with HIV, including enhanced quality of life and decrease in morbidity and mortality [33]. Achieving the 95-95-95 targets would also have the benefits on reducing transmission that have been described above. Indeed, all persons living with HIV are entitled to access to ART, as all have an equal right to live healthy lives. Ensuring access to HIV treatment for PLHIV is a concrete action in addressing inequalities within the global AIDS response, to further fulfil the rights to health for all.

## Conclusions

The overall vision for the global HIV response includes reducing new infections, stigma and discrimination and mortality due to HIV. The new targets are directly related to reducing inequalities, which can only be achieved if societal barriers that perpetuate stigma are overcome including with fully resourced community-led programming.

The ambition of the 95-95-95 targets for all relevant populations signals the world’s continued commitment to the AIDS response and to achieving the 2030 sustainable development goal of “ending AIDS” as a public health threat. They provide an ambitious agenda which address inequalities in HIV treatment outcomes and a vision for national AIDS responses. Closing the gaps and disparities in testing and treatment coverage across countries, sub-national areas and population groups will ensure the population-level declines in HIV incidence necessary to end AIDS by 2030 and will ensure equity in the global AIDS response.

## Supporting information

S1 Data (XLSX)

S2 Data (XLSX)

S3 Data (XLSX)

S4 Data (XLSX)

S5 Data (XLSX)

S1 Annex (DOCX)

S2 Annex (DOCX)
