# Correction: Achieving the 95 95 95 targets for all: A pathway to ending AIDS

**DOI:** 10.1371/journal.pone.0318123

**Published:** 2025-01-24

**Authors:** The PLOS ONE Staff

## Notice of Republication

This article was republished on January 13, 2025, to correct errors in the author list and author affiliations that were introduced during the typesetting process. The publisher apologizes for the errors. Please download this article again to view the correct version. The originally published, uncorrected article and the republished, corrected articles are provided here for reference.

## Supporting information

S1 File Originally published, uncorrected article. (PDF)

S2 File Republished, corrected article. (PDF)
