# Supplementary material for: Achieving the 95 95 95 targets for all: A pathway to ending AIDS
Source: PLoS One. 2022 Aug 4;17(8):e0272405. doi: 10.1371/journal.pone.0272405 (PMC9352102; doi:10.1371/journal.pone.0272405)
Supplement: S1 Annex — (DOCX) [file pone.0272405.s006.docx]

**Annex 1**

**Table 1: Examples of countries with diverse HIV epidemics that have made substantial progress in testing and treatment for PLWH**

| **Country** | **Achievement against the target of 90-90-90 by 2020** ^a^ | **2020 HIV Prevalence**^b^ | **GDP per capita (constant 2010 US $)** | **World Bank Income Group** |
| --- | --- | --- | --- | --- |
| Eswatini | >95 - >98- 94 | 26.8 [25.4 - 28.1] | 4 690.2 | Upper Middle |
| Switzerland | 93- >98- 96 | 0.2 [0.1 - 0.2] | 80 132.4 | High |
| Rwanda | 92->98-96 | 2.5 [2.3 - 2.7] | 849.0 | Low |
| Qatar | 93->98-96 | <0.1 [<0.1 - 0.1] | 59 924.0 | High |
| Botswana | 91-95->98 | 19.9 [18.2 - 21.0] | 7 306.5 | Upper Middle |
| Slovenia | 90-96-95 | <0.1 [<0.1 - <0.1] | 25 759.2 | High |
| Uganda | 91->98-90 | 5.4 [5.1 - 5.8] | 958.2 | Low |
| Malawi | 91-94-93 | 8.1 [7.6 - 8.5] | 526.9 | Low |

^a^ UNAIDS Special Analysis, 2021.

^b^ UNAIDS Estimates, 2021. Point estimate and uncertainty bounds.
