# Supplementary material for: Achieving the 95 95 95 targets for all: A pathway to ending AIDS
Source: PLoS One. 2022 Aug 4;17(8):e0272405. doi: 10.1371/journal.pone.0272405 (PMC9352102; doi:10.1371/journal.pone.0272405)
Supplement: S2 Annex — (DOCX) [file pone.0272405.s007.docx]

**Annex 2**

**Fig 7. Regional incidence to prevalence ratio among adults 15+ and ART coverage, 2000-2020**

**
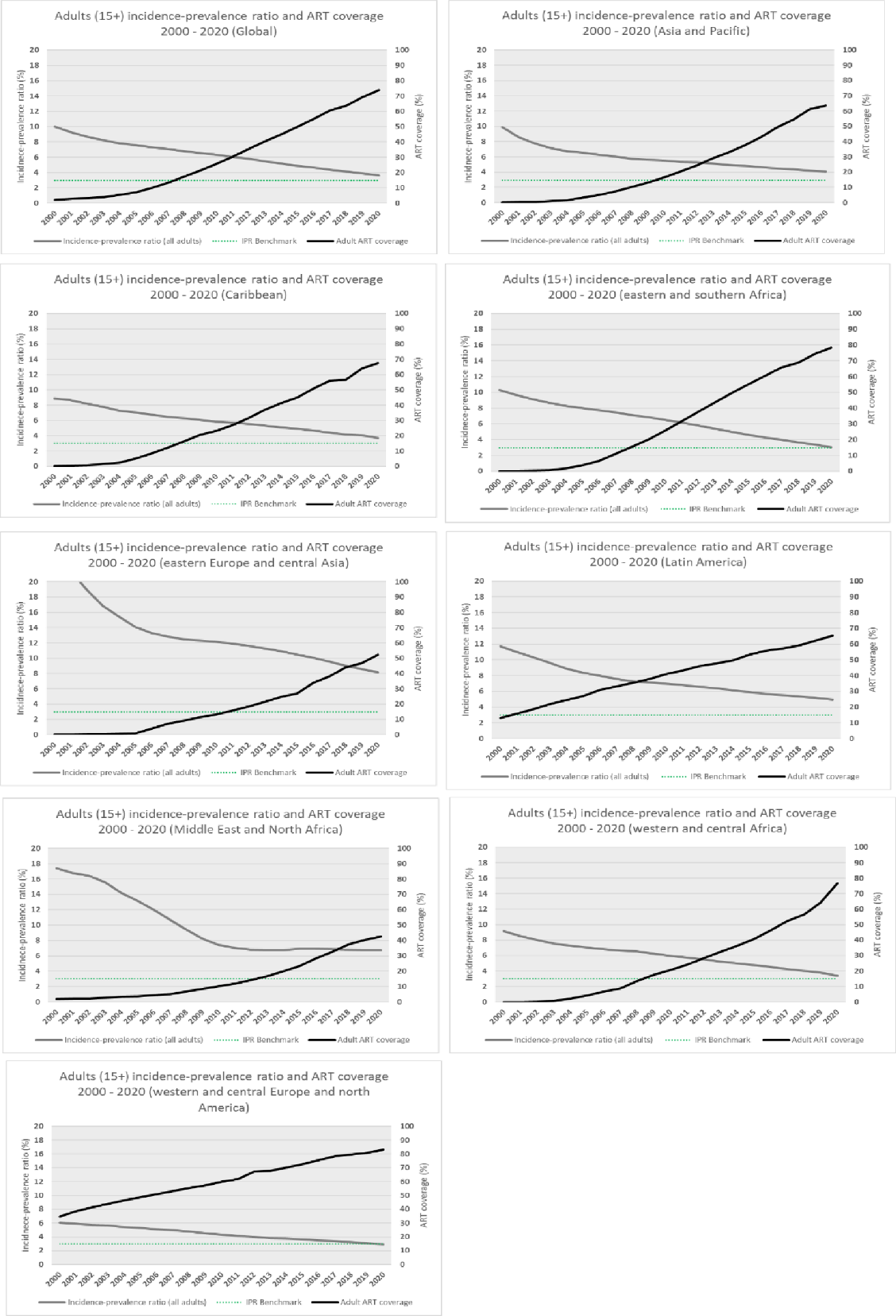
**

UNAIDS Special Analysis, 2021
